# “I just have to hope that this abortion should go well”: Perceptions, fears, and experiences of abortion clients in Nigeria

**DOI:** 10.1371/journal.pone.0263072

**Published:** 2022-02-07

**Authors:** Anna J. Katz, Ana Maria Ramirez, Chiara Bercu, Sofia Filippa, Osasuyi Dirisu, Ijeoma Egwuatu, Sybil Nmezi, Lucky Palmer, Sarah E. Baum

**Affiliations:** 1 Ibis Reproductive Health, Oakland, CA, United States of America; 2 University of California, Berkeley, School of Law, Berkeley, CA, United States of America; 3 Generation Initiative for Women and Youth Network, Lagos, Nigeria; 4 Ipas Nigeria, Lagos, Nigeria; Vital Strategies, UNITED STATES

## Abstract

This qualitative study aimed to examine how abortion clients in Nigeria perceive abortion and explore the role their beliefs and fears play in their care-seeking experiences and interactions with providers. Abortion is severely legally restricted in Nigeria but remains common. We conducted in-depth interviews with 25 people who obtained abortion services through three distinct models of care. We coded interview transcripts and conducted thematic analysis. Clients perceived negative attitudes toward abortion in their communities, though clients’ own beliefs were more nuanced. Clients recounted a range of fears, and nearly all mentioned worrying that they might die as a result of their abortion. Despite their concerns, clients relied on social networks and word-of-mouth recommendations to identify providers they perceived as trustworthy and safe. Kind and non-judgmental treatment, clear instructions, open communication, and reassurance of privacy and confidentiality by providers alleviated client fears and helped clients feel supported throughout their abortion process. Within restrictive contexts, the mobilization of information networks, provision of high-quality care through innovative models, and personalization of care to individual needs can assuage fears and contribute to reducing stigma and increasing access to safe abortion services.

## Introduction

Induced abortion is common globally, with an estimated one in four pregnancies ending in abortion, and has been affirmed as a human right [1, 2]. Yet 450 million women—representing 27 percent of women of reproductive age worldwide—live under the most restrictive categories of laws that limit their ability to exercise their right to abortion, permitting abortion only to save the pregnant person’s life or prohibiting abortion altogether [3]. There are 18 countries throughout the African region with restrictive laws in these categories [3]. In Nigeria, the penal and criminal codes prohibit abortion in all cases except when performed to save the life of the pregnant person and subject abortion seekers, and those who assist them, to heavy penalties including life imprisonment [4, 5].

In such restrictive settings, and even in environments with more liberal abortion laws, a range of structural factors such as cost, distance, provider attitudes, and privacy concerns shape and often limit people’s ability to access safe, timely care [6–8]. In addition, people’s access to abortion services is mediated by their knowledge or perception of the abortion law, which is often poor [9]. These barriers are compounded by legal restrictions, pushing people toward abortion methods that may be unsafe. Unsafe abortion and related mortality are consequently highest in countries with few avenues for legal abortion [10].

Strategies for increasing abortion safety and reducing abortion-related morbidity and mortality have emerged in restrictive legal settings around the globe. People seeking abortion can turn to clinics that offer information and support through harm-reduction models and millions of people seek post-abortion care for abortion-related complications each year [11, 12]. Medication abortion provision through pharmacies and drug sellers is also common, including in legally restrictive settings, though knowledge of effective regimens among pharmacy workers is often limited [13]. In one study, the availability of misoprostol tended to increase the safety of abortion, but such progress was constrained by individuals not having accurate information on how to safely self-manage an abortion using misoprostol [14]. Lastly, alternative models of abortion care, such as hotlines and community pharmacies, have emerged in restrictive legal settings to provide accompaniment and support during self-management of medication abortion [9]. Informed by mounting evidence that people can safely and effectively self-manage their abortions with misoprostol or a combination of mifepristone and misoprostol [15–17], some organizations work outside of the formal healthcare system to provide information, counselling, and support to individuals considering medication abortion [11, 18, 19].

In Nigeria, an estimated 1.25 million induced abortions occurred in 2012 at a rate of 33 abortions per 1,000 women aged 15–49 [5]. A more recent, nationally representative survey of women of reproductive age found a rate of 29 abortions per 1,000 women aged 15–49 [20]. Most of these abortions are performed clandestinely, by unskilled providers, or both [4, 21]. As a result, unsafe abortion remains a major contributor to maternal morbidity and mortality in Nigeria, which has one of the highest maternal mortality ratios in the world [22]. Beyond safety concerns, the clandestine nature of abortion means that people seeking abortion services may be financially exploited by abortion providers and avoid seeking hospital-based care unless absolutely necessary [21]. In addition, a recent study documented high levels of abortion stigma among close to half of Nigerian abortion seekers [23].

Abortion stigma, defined as “a shared understanding that abortion is morally wrong and/or socially unacceptable,” and the social norms governing pregnancy and reproduction likely shape attitudes toward abortion at the individual and community level [24]. Abortion stigma is most pronounced in countries with highly restrictive abortion laws, including Nigeria [25]. Research has also shown a strong connection between abortion attitudes and religion or religiosity, including in sub-Saharan African countries [26–28]. People’s beliefs about abortion and the social norms that are pervasive in their communities can influence their decisions about care-seeking. For example, women in Ghana who sought care outside the formal health care system, despite laws permitting abortion, reported fears of judgement and mistreatment by health workers and a desire to keep the pregnancy and abortion a secret as motivating factors in their decision[29]. Studies have similarly documented that abortion-seekers may choose informal sector or less-safe abortion services that they perceive as more private, rather than risk the possible exposure and social stigmatization that may result from seeking care from high-profile health facilities [30–32]. In Zambia, a study found that the advice and knowledge of close confidants and community members influenced people’s pathways to abortion care and choice of service provider, such as through insider knowledge of the healthcare system [33].

This qualitative study aimed to examine how abortion clients from a range of care models in Nigeria perceive abortion and explore the role their beliefs and fears play in their care-seeking experiences and interactions with providers. The perspectives from clients themselves are critical in order to identify interventions to better support people in their pathway to abortion, especially in highly restrictive settings.

## Methods

Between December 2018 and February 2019, we conducted semi-structured, in-depth interviews with clients who had an abortion in Nigeria as part of a larger, multi-country study [34]. The larger study aimed to assess client perspectives on abortion quality of care in four countries: Argentina, Bangladesh, Ethiopia, and Nigeria. In each country, clients were recruited from a range of service delivery models in an effort to capture some of the diversity of abortion experiences. We sought to recruit approximately 100 abortion clients across the four countries, with approximately 25 participants in each location.

In Nigeria, we recruited abortion clients in Lagos state and Ogun state from two healthcare clinics, a safe abortion hotline, and four proprietary and patent medicine vendors (PPMVs). The private healthcare clinics provide procedural abortions despite the legal restrictions; clients return to the clinic for follow-up care if needed or desired. The safe abortion hotline offers free and reliable information on reproductive health, including pregnancy, abortion, and post-abortion care. When clients call the hotline, trained volunteers provide confidential information and support, including how to safely use abortive medication. Clients often make multiple calls throughout the course of their abortion care. PPMVs are owner-operated drug retail outlets that sell medicines and provide services for a variety of health needs, including malaria, common cough and cold, and reproductive health [35]. Owners often work out of their home or garage. Though PPMVs are not legally authorized to provide abortion services, people consider them a source of both information and medication for people seeking abortion [36]. We identified each recruitment site based on the expertise and relationships of our local partners. Participants were recruited by trained personnel from each recruitment site.

We developed interview guides that were similar for each country in the larger study and adapted the guides with input from local study teams. The instrument in Nigeria incorporated open-ended questions exploring participants’ knowledge, perceptions, and beliefs related to abortion; expectations and fears prior to obtaining care; experiences accessing and receiving abortion services; and priorities for high quality abortion care. We also asked questions designed to capture clients’ perception of social norms and abortion-related stigma.

Clients were invited to participate in the study shortly after their service. Healthcare clinic clients were invited to participate after receiving a manual vacuum aspiration (MVA) or during the follow-up visit after a medication abortion. Clients who received care through accompaniment with the safe abortion hotline were invited to participate in the study either at the end of their follow up call or when contacted within three months of their abortion. Local study staff randomly selected PPMV clients from lists of people who had obtained an abortion within the past three months and invited them to participate in the study. To ensure confidentiality, clients were recruited by service providers who already knew that the client was seeking or had obtained abortion care. Recruitment staff informed all potential participants that their decision to participate or not participate in the study would in no way affect their care. Clients were eligible for the study if they were at least 15 years old, spoke a study language (English, Pidgin English, or Yoruba), and had had an abortion within three months prior to recruitment.

All local study staff participated in in-person trainings to discuss the instrument and receive information on the research objectives, study methodology, probing techniques, and ethical guidelines. The study coordinator was trained in qualitative methods and interviewing techniques, had extensive experience conducting research with stigmatized populations, and lived and worked in the region where the study was conducted. She carried out two pilot interviews to test comprehensibility and adjust questions or probes where necessary. The study coordinator and one additional interviewer, neither of whom were involved in the participants’ care, used the revised instrument to conduct all interviews in a private space at or near the service provider or over the telephone. The interviewers reviewed the consent form and obtained verbal consent to conduct and audio-record the interview. Participants received ₦3,000 in cash or mobile money (approximately $8.25 USD at the time of data collection) for their time and transportation. This study received ethical approval from Federal Medical Centre (Nigeria) and Allendale Investigational Review Board (USA).

Interviews were professionally transcribed in the language in which they were conducted; five interviews were translated from Yoruba to English for analysis. The research team developed an initial codebook for the larger study with *a priori* codes based on the interview guide and quality of care frameworks in reproductive and maternity care. We also added codes based on emerging themes in the interviews in all four countries. Pairs of researchers then coded two transcripts from each country and met to discuss discrepancies and refine the codebook. All transcripts were coded with the final codebook by two researchers using MAXQDA 2018 qualitative analysis software (VERBI Software 2019). We double-coded approximately 20% of all the dataset in order to assess reliability in the coding across researchers. For the analysis among the sample in Nigeria, we assessed key themes in the data with a focus on beliefs, fears, and experiences, while also considering patterns among people who had prior abortions. We present findings that emerged across the sample and highlight distinctions by model of service delivery when relevant. Quotes are identified by participant age and service delivery model.

## Results

We conducted in-depth interviews with 25 abortion clients who obtained abortion care or support from healthcare clinics (n = 10), a safe abortion hotline (n = 10), and PPMVs (n = 5) in two states in Nigeria. The mean age of participants was 25 years, with a range of 16–41 years. Half of the participants had one or more children and nearly two-thirds were unmarried. Fifteen participants—all of those recruited through the safe abortion hotline and PPMVs—had a medication abortion, and all 10 clients recruited from clinics had a procedural abortion. Nine participants reported having at least one prior abortion. These data are summarized in Table 1.

**Table 1:** Participant characteristics.

	Total
	n = 25
**Age (years)**	
Mean	25.44
16–24	16
25–35	5
> 35	4
**Marital status**	
Married	9
Unmarried	16
**Number of children**	
0	13
1–2	8
3 or more	4
**Prior abortions**	
No	16
Yes	9
**Type of abortion**	
Medication	15
Procedural	10
**Service-delivery model**	
Healthcare clinic	10
Safe abortion hotline	10
PPMV^a^	5

^**a**^ Proprietary and patent medicine vendors are regulated owner-operated drug retail outlets that provide a range of medications and general health services.

### Client perceptions of abortion

Most clients perceived negative and condemnatory attitudes toward abortion in their communities. Participants explained that abortion is generally viewed as an immoral act; many stated that people who have abortions may face exclusion, judgment, and shame in their families and social circles. “I don’t think anybody will accept for you to have an abortion,” one client explained, “Everybody sees it as a great sin in Nigeria” (Age 16, hotline). Another participant described how this abortion stigma could manifest:

People will call you all sorts of names and say this woman have [sic] been committing abortion of her pregnancies, you are not a good person. If you commit abortion, you are not a good person, your background is poor, maybe your parents didn’t train you well and all that. (Age 24, hotline)

Participants spoke about the silence and secrecy that shrouds abortion in their communities and strongly emphasized that individuals who have abortions limit disclosure. “Nobody talks about doing an abortion, so there is no social consequence. Except [if] you say you did an abortion,” stated one client. “It can’t be a public thing. So, it is permanently hush” (Age 24, hotline). Such a culture of secrecy may be a product of, or exacerbated by, the legal status and perceived illegality of abortion in Nigeria. Five participants confidently reported that abortion is illegal in Nigeria, and while the majority of participants expressed uncertainty about abortion’s legal status, they still voiced the perception that abortion is criminal or otherwise taboo. For example,

I think abortion is illegal in some countries, I think in Nigeria too it’s illegal to do abortion. But I’ve not really heard that someone committed abortion and she was arrested for that or anything… But I think it’s illegal, illegal yes… (Age 23, hotline)

Half of participants invoked religion, both when speaking about their own beliefs and when describing opposition to abortion in religious texts and places of worship. Clients described abortion as “a sin,” “killing,” and “taking another person’s life” and highlighted the anti-abortion stance of their church or mosque. To have an abortion is to go against the religious doctrine of their faith, participants explained, and individuals who are known to have abortions may face exclusion from religious activities or be forced to repent. For some participants, their religious beliefs clashed with their decision to have an abortion. One participant stated,

I am a Christian; I know what I am doing today is against the doctrine of the Christian[s] but I don’t have any other choice than what I have done today… [What] I do today, I know it’s not right. (Age 36, clinic)

Like this client, several participants talked about internal conflict or self-judgment related to their abortion, explaining that “the guilt of abortion never leaves you.” However, others expressed a range of personal beliefs that deviated from what they perceived as the mainstream views in their community. Several clients spoke about abortion as an individual choice or articulated circumstances under which abortion is acceptable. Though her church preaches that abortion is forbidden, a 23-year-old hotline client articulated her own opinion, explaining, “I believe that any unwanted pregnancy should be removed as far as you call it unwanted. Whatever you don’t want, you remove it. That’s my belief. I don’t see anything wrong with that.”

Another client described how her views about abortion had developed over time:

A woman can have an abortion when she wants, that is not bad at all. Before I had this information, especially with the kind of church that I go to, they preach that abortion is not good, it is a sin against man and God. But when I, over time, I now started hearing the other side of abortion, which I so much believe now. (Age 24, hotline)

Clients spoke about the ways that abortion creates an opportunity for young people to continue their education and enables mothers to better care for their existing children. Another participant spoke about weighing the life and needs of the pregnant person against the potential life of the fetus:

I think I have always just felt like it is your life, do what you want to do with your life generally. However, it is supposed to be another life as well, right, but given that the life isn’t out yet… I think you have to make a choice for both of you. So if you can take care of the child, why not, but if you can’t, why should you? (Age 24, hotline)

Participants also maintained that abortion is both common and “normal,” despite perceiving abortion stigma in their communities, and many participants knew someone personally who had had an abortion. “[A]bortion is not good,” one client explained, “but there is not any woman who has said they’ve never do [sic] it before. It’s a, it’s a normal thing” (Age 26, clinic).

### Fears prior to seeking abortion

All participants recounted the fears that they held while considering abortion and seeking abortion care, including pain, infertility, incomplete abortion, and death. Nearly every client interviewed reported feeling afraid that she might die as the result of her abortion. Participants described worrying that they would bleed to death, foregoing general anesthesia out of fear that they would die while unconscious, and praying that they survive the abortion. “I was afraid of having complications that results [sic] in death,” one participant explained, “and I started making resolutions that if I survived this experience, I will never attempt it again” (Age 39, clinic).

Many clients spoke about the stories and rumors that fueled their fears. Almost all clients knew personally or had heard of someone who died or became infertile as a result of their abortion, either through their social circles or the media. One client summarized the pervasive narrative in her community that frames abortion as dangerous and sinful: “You died, the child died, you bleed to death, you go to hell. So that’s what you hear generally…you hear that everyone that does abortion dies or the person can never give birth again” (Age 24, hotline).

Infertility, often described as losing or injuring the womb, was another prevailing concern among participants. A 36-year-old participant who had a clinic-based abortion recalled fearing, prior to her first abortion, that she might be punished by losing her ability to have children: “[Y]ou know it’s not right [to have an abortion] and you still went ahead to do it, you might have the punishment for it. Maybe you might not be able to have another child. That’s your punishment.” Several participants understood infertility as a consequence for violating moral norms by having an abortion. Another client described the fears she held about her future fertility:

Some people said you might not give birth again, some people said you might not conceive again after it. That was what I know. I just have to give God, I just have to hope that this abortion should go well. (Age 17, hotline)

When reflecting on how her actual experience compared to her expectations, another participant said,

Aaaah, you see it was like the direct opposite. I was thinking I was going to die and I would have probably lost my womb but then I’m alive, fine, and I’m still menstruating meaning my womb is still [intact]. (Age 19, PPMV)

While fears of death and infertility were principal, clients also reported fearing pain, bleeding, and other side effects, including the possibility of contracting HIV or another infection from unsterilized instruments. Participants who had procedural abortions more frequently described fears of pain, bleeding, and infection than those who obtained medication abortions. Several procedural abortion clients also described fears about the procedure itself, the tools that would be used, and their level of awareness during the procedure. Rather than being unique to abortion care, though, these concerns seemed to reflect a fear of surgery broadly, including worries about being anesthetized and having their bodies “opened.” One client perceived similarities between abortion and other surgical procedures that she found concerning: “Usually my major fear is the sight of the instruments… Looking at the instruments they are no different from the one used in the event of major surgery” (Age 41, clinic).

Clients not only worried about abortion safety and side effects, but also reported concerns related to abortion effectiveness. Several participants, particularly young clients, feared that they would not successfully terminate the pregnancy. “My biggest fear was like, will this really work? That was my fear,” reported a 23-year-old hotline client. Fears related to being judged by a provider while seeking care or being denied an abortion service outright were similarly salient among young participants. Another hotline client, also 23 years old, shared a story of her friend being chased out of a pharmacy after attempting to procure medication abortion pills for her own abortion. Familial judgment was a concern among a minority of young people, too, as several clients reported worrying that their family would find out about their pregnancy or abortion.

Among participants who had had prior abortions, the majority described fears that seemed informed by their negative past experiences. Clients described having clandestine procedural abortions performed without pain medication, experiencing unbearable pain, and passing the products of conception days after an incomplete procedure. One participant was abused by a relative and forced to ingest an abortive substance until she miscarried the pregnancy. Despite having had and survived an abortion in the past, these participants often remained fearful of the judgment, pain, and potential complications their abortion might bring. Two clients, however, only discussed the fears they held prior to their first abortion; they returned to the same provider for subsequent abortions and did not express concerns specific to those abortions.

### “Just summon my courage”: Seeking abortion care in the face of stigma and fear

In deciding where and from whom to seek care, clients prioritized a provider who they perceived as legitimate or a source of physically safe care and who they believed would offer a confidential abortion service. Choosing a provider with these qualities helped combat client fears. One participant explained,

You might die, you might do this, you might not be able to give birth to another child, blah, blah, blah, you lose your womb whatever. As for me, I think when you have a good practitioner like [clinic name], you are safe. (Age 32, clinic)

This participant felt confident that the provider offered safe abortion services because she had visited the clinic for contraceptive care and observed that the waiting room was often full. “If he is not good, people will not be coming in here,” she said, “because there are lots of quacks around” (Age 32, clinic). Several participants made this distinction between legitimate providers and “quacks”—individuals offering fraudulent or substandard medical care—often based on other people’s experiences with post-abortion side effects and complications. They noted that individuals who seek care from so-called “quacks” suffered from side effects and “regret it,” but for those who obtain services from a qualified provider “there won’t be any problem.”

For some clients, believing that a provider would maintain their confidentiality was as important as identifying a provider who was technically skilled. One client emphasized the importance of private care:

I needed something that was kind of private, confidential, like I didn’t want everybody knowing about my business… I knew I didn’t just want to do anything that would involve other people I knew, so I just had to like go through [the hotline] because I know they are quiet and confidential and safe. (Age 17, hotline)

To identify these safe abortion providers, clients relied on close social networks and word-of-mouth recommendations. Clients often chose their provider based on the recommendation of a friend, partner, or family member who assured them that they would receive a safe abortion and quality care. For example,

When [I] told my sister that it has happened, that I am pregnant…she said I can take you to a hospital. I said where, she said [clinic name], their work is perfectly ok, that she has come here too before, that it was perfectly ok. She gave me the assurance, she gave me the 100% that it will be perfect. (Age 24, clinic)

These testimonials helped reassure clients and encourage them to seek care. “I trust my friend but I know she is not a person that can lead someone astray… So, I decide to follow her advice and call the hotline,” said one client (Age 23, hotline). “[My friend] told me when she done [sic] her own, it is very easy and she didn’t have anything and there is no complication after she done it,” another participant explained. “I think that’s why I summon the courage the first time I was here to come here” (Age 36, clinic). Like this participant, who alludes to her first abortion, some clients returned to providers they already had a relationship with, whether from receiving services (including for abortion and childbirth) or because the provider was a relative, family friend, or neighbor. Like choosing a provider recommended by a friend, seeking care from someone that they knew and trusted helped clients feel safe while accessing abortion care.

While personal relationships and provider recommendations eased participant anxiety, clients made their care-seeking decisions within a context of scarcity. A few clients only contacted their health care provider after considering or attempting to self-manage their own abortion. One participant recalled using a “native medicine” and contacting the hotline when it did not successfully terminate her pregnancy. Another client considered obtaining an abortion in her friend’s home, but instead decided to “come to a proper place and have it done.”

Clients commented on not having many options when seekingabortion care, and some conveyed a sense of urgency or desperation. As one participant stated, “The reason I came to [clinic name] is because I do not have any alternative” (Age 41, clinic). Clients also described weighing their fears and the risk of having an abortion against the risk of having a child. “I was afraid I would die if I had an abortion,” one explained, “[but] I just had to choose. I had to do what, what could give me another chance. I needed another chance, that’s why I went through with it” (Age 19, PPMV). Though they worried about the potential consequences of having an abortion, from social exclusion to lasting morbidity, almost all participants felt certain about their decision not to continue the pregnancy. One participant explained that having an abortion was her only option, despite the risk:

I decided [to have an abortion] because I was, I didn’t have any other alternative…having known that I cannot keep this baby. The man that is responsible is not really ready for that, so I didn’t have any other alternative than to take risk. (Age 23, hotline)

### “That fear disappeared”: Provider reassurance and support

Many participants highlighted ways that providers assuaged their fears and provided comfort, reassurance, and support. The key themes were: kind, caring, and non-judgmental treatment; clear instructions and understandable information; open and ongoing communication; and private, confidential care.

#### Kind, caring, and non-judgmental treatment

From their first encounter with clinic, hotline, or PPMV staff, clients described being welcomed with warm, non-judgmental care, which helped them relax and feel comfortable. One client recounted her experience arriving at the clinic and waiting for care:

On arrival the staff will first ask you your mission in the hospital and oftentimes make out time to exchange jokes and pleasantries with you and whatever we need before the arrival of the doctor is given to us. They are neither harsh nor rude to us… Their attitude your attitude makes me so comfortable that each time I want to come here I have no fear or ill feelings. (Age 39, clinic)

A hotline client described hesitating to call the hotline out of fear of judgment; she worried that the counselor “will just be like other people…before you know it, they will start shouting.” But when the hotline counselor answered the call with kindness and warmth, the participant felt comfortable sharing her situation: “Immediately I found out that the person was very nice. I just opened up to the person…I feel very relaxed, I am very relaxed. The way they spoke to me. I was very, very happy.” Another client, who had experienced complications from an incomplete procedural abortion in the past, alluded to judgmental providers: “[T]here are some places you will go and they will be harsh in their treatment of you” (Age 41, clinic). She appreciated that the abortion clinic staff treated her well, like a customer; the experience felt like a normal exchange of goods and services. When asked how she would describe her provider to a friend, one participant replied, “I would say she is someone very welcoming and warm, she would make you feel like even if you are doing the wrong thing, it is very right” (Age 19, PPMV).

In some instances, clients felt that provider kindness and care far exceeded their expectations. One participant explained that her provider volunteered to drive her home at the end of the day as she could not drive herself after her procedure; it made her feel “like a princess” (Age 32, clinic). A hotline client explained how the counselor supported her through multiple attempts to obtain misoprostol from pharmacies.

#### Clear instructions and understandable information

Participants described how providers shared explanations of the entire abortion process and gave clients step-by-step instructions, which helped alleviate client fears and helped clients feel safe. “So, they started telling me everything, what to do and how to do it and the kind of things I will see,” one participant recalled. This detailed counselling helped her feel safe, relaxed, and prepared for the abortion:

Yes, they really prepared me, even, they made me have confidence to know that yes, ok, after this this is the next thing. Ok, look at what to do, yes, ok… I had a very big confidence through them that nothing would happen. (Age 23, hotline)

Though such step-by-step instructions were most common among hotline clients, who also received guidance on how to obtain misoprostol without disclosing their plans to terminate their pregnancies and information on seeking follow-up care, clinic- and PPMV-based providers also shared information that helped minimize client fears. “She prepared my mind very good, because she explain [sic] to me and told me the side effects, everything,” one participant said of her PPMV provider. One clinic-based client appreciated the opportunity to share her fears with her provider and ask questions:

I have often heard some complain about the side effects such as excessive bleeding after the procedure and this sometimes results in dizziness. I shared these fears with the healthcare provider who made efforts to allay my fears by explaining that this may be as a result of the person who conducted the procedure. (Age 39, clinic)

While providers usually shared information that lessened client fears, one participant reported that the information about what to expect after taking misoprostol made her feel somewhat scared. Nevertheless, she found the counselor’s detailed instructions and information “very helpful” (Age 16, hotline).

#### Open and ongoing communication

Hotline and PPMV clients, who self-managed their abortions with provider guidance, valued the ability to communicate with providers during and after their abortions for ongoing information and support. Participants described calling or returning to their providers to seek additional guidance or ask clarification questions, which helped them feel confident throughout the abortion process. “I was having kind of some pain, so I just wanted to be assured that it was normal,” one client explained, articulating her desire for reassurance (Age 17, hotline).

Hotline and PPMV staff would also call clients to check in on them, making clients feel relaxed and cared for. “They were like checking on me as a doctor you know, but it made me, the thing made me to relax my mind,” one client explained. The follow-up calls alleviated her fears as well: “I was not even afraid of anything again because, I know that in a day or two days, they would call me back to ask me, ‘How is it? How are you? How is it?’ So I was just happy about everything” (Age 18, hotline). Another client explained how the provider reaching out made the hotline feel more personal than other models of abortion care:

[H]ad it been I went to a doctor or any pharmacy or one village and get some herbs, nobody will called to know how you were feeling. They will feel like I’m done with you, go and do whatever you see it’s okay, but for them [the hotline] to call back and check on me it’s so good, I really appreciate. (Age 23, hotline)

#### Reassurance of privacy and confidentiality

Though participants expressed concerns related to the privacy of their abortion care, providers helped them feel more at ease and unafraid by assuring them that their abortion would remain a secret. “The time I first met him, I just have to come out for him [and ask] am I safe?” said one client. “Is it confidential? Would there be any police report? … But he said, no, that I am safe. So that’s why I just go with him that time” (Age 36, clinic).

Participants recounted the importance of a private location for their abortion. One client described staying at the PPMV while she completed her abortion, stating, “[The provider] said I was going to see blood and I couldn’t go home because I don’t want my mummy to notice” (Age 19, PPMV). In contrast, a client who obtained an in-clinic abortion reported feeling satisfied with the level of privacy she received, even though she left the clinic while feeling dizzy and weak shortly after the procedure because she was worried of being seen by someone she knew. This suggests that the provider could have done more to provide a private space for recovery.

Some participants also felt protected by the hotline model of service delivery, which did not involve any in-person interaction. “I felt safe, I felt protected, I felt the whole thing was being done confidentially so just between me and [the provider], no other person, no other third-party was there, was involved,” one client said (Age 17, hotline). Hotline clients appreciated that they only interacted with providers via phone and that they did not need to provide much personal information in order to receive care.

## Discussion

This qualitative study provides insight into clients’ perceptions and fears related to abortion and experiences seeking care in two states in Nigeria. People who obtained abortion services through three distinct models of service provision in this restrictive legal context largely relied on the advice of trusted community members to identify safe abortion options. Clients turned to members of their social networks, whose knowledge of particular providers and testimony about the safety and quality of their services helped encourage clients to seek care and lessened client fears. This finding parallels a study in Zambia, where participants recruited from a public hospital also highlighted the influence that trusted individuals had on both the provider they chose and their pathway to care [33]. The important role that social networks played in identifying safe abortion options suggests that people in communities with less knowledge about abortion or higher levels of abortion stigma, such as young people or individuals in insular religious communities [37], may have a harder time accessing safe care. People seeking abortion in Nigeria may not know about safe options like hotlines where they can obtain accurate information and support. A growing body of evidence suggests that people have safe, effective, and acceptable abortions with such providers in West Africa and other contexts [11, 38, 39]. Participants’ reliance on word-of-mouth recommendations presents a possible intervention point for increasing access to services and mitigating abortion-related fears. Programs that train community volunteers to disseminate information about sexual and reproductive healthcare, including abortion, have effectively increased knowledge of safe abortion options elsewhere [40] and may be a powerful way to mobilize community-based networks in Nigeria. There is also growing evidence that digital media campaigns can be an effective platform for SRH promotion, particularly for young people [41], and may provide another avenue for disseminating abortion-related information and connecting people with safe abortion providers. In addition, given that many clients perceived abortion as completely illegal, despite the life endangerment exception, these programs could be harnessed to increase access to information about the legal status of abortion in Nigeria.

Our findings also identify four elements of quality abortion care that reassured clients and helped them feel supported throughout their abortion process: kind and non-judgmental treatment, clear and understandable information, open and ongoing communication, and reassurance of privacy and confidentiality. A study of Nigerian and Ugandan women’s perspectives on quality of care during childbirth identified similar domains of high-quality care, including treating clients with respect and empathy, building rapport, using clear language, and protecting privacy, which indicates interesting parallels in person-centered care in childbirth and abortion [42]. Other studies of abortion quality have similarly documented the importance of interpersonal treatment by providers [43, 44], detailed information provision [45, 46], and a private and confidential service [30, 43]. Alternative models of abortion care may be particularly poised to meet clients’ needs in these domains, especially in restrictive legal contexts. Safe abortion hotlines, for example, provide a naturally private service by eliminating any in-person interaction and allow clients to get in touch virtually with their counselor for information and support throughout the abortion process. It is crucial to develop channels of communication throughout the course of the abortion experience for clients, particularly if they obtain medications and complete their abortion at home. Messaging technologies like Signal and WhatsApp, which provide an encrypted platform for instant communication, may be one solution.

This study highlighted the ways in which client expectations and fears for care—and therefore what they prioritize in an abortion service—are context specific. Given the stigma surrounding abortion in Nigeria [23, 47], these findings demonstrate some of the ways that stigma can manifest for abortion clients—including through concerns about judgment, feelings of guilt, and prevailing narratives of abortion as dangerous or deadly—and have implications for service delivery. Previous research has illustrated the ways that social norms and stigma contribute to low expectations among abortion-seekers and highlighted the potential relationship between expectations and perceptions of quality [48]. People who have low expectations for abortion services may be more likely to report high levels of satisfaction or that their experience exceeded their expectations. This may not reflect the quality of the service itself as much as the satisfaction or relief of receiving a wanted abortion [48, 49]. Researchers, health facilities, and governments who are monitoring and evaluating abortion quality should consider universal minimum standards while also developing guidelines for counseling and interpersonal interactions that are specific to the context in which they provide care. For example, whereas providers in countries where abortion is less stigmatized may not need to assuage clients’ fears of abortion morbidity, providers in Nigeria could consider addressing unsafe abortion and abortion-related complications during counseling in an effort to combat stigmatizing narratives and alleviate widespread fears. In order to deliver truly person-centered and high-quality care, abortion providers and service delivery models must take into account the environment in which they are providing services and the needs of individual clients.

This study contributes a range of perspectives from abortion clients in Nigeria; however, there are several limitations. While we aimed to understand clients’ experiences of quality of care across multiple service delivery types, the perspectives presented here do not necessarily represent all abortion seekers in Nigeria, including those who wanted but were unable to obtain an abortion, those who used traditional or herbal abortion methods, and those who self-managed their abortion without the support of a provider or counselor. In addition, our sampling did not intend to facilitate comparisons between service delivery models, or based on financial or social support circumstances, so we are unable to address the impact of these factors on fears or assessment of quality of care. Finally, clients who had better abortion experiences may be more likely to choose to participate, or service delivery sites that offer higher quality care may have been more likely to agree to serve as recruitment sites. Therefore, we may not have heard from clients with the most negative experiences.

## Conclusion

These findings help illustrate how the perceptions and fears of abortion clients inform their experiences seeking and obtaining care in Nigeria. Though abortion clients described negative attitudes toward abortion in their communities and held many fears, including of judgment, infertility, and death, they leveraged their social networks to identify trustworthy providers. Providers play a key role in addressing abortion misconceptions and fears among their clients and counselling guidelines should continue to center the individual needs and concerns of each patient. Efforts to improve abortion quality of care in Nigeria should emphasize interpersonal domains such as non-judgmental care, open communication and confidentiality. Alternative models of abortion care, such as safe abortion hotlines and community pharmacies, may be particularly poised to increase abortion access and provide high-quality care in restrictive legal and social contexts as well as around the globe.

## Supporting information

S1 File Interview guide. (DOCX)
