# Supplementary material for: “I just have to hope that this abortion should go well”: Perceptions, fears, and experiences of abortion clients in Nigeria
Source: PLoS One. 2022 Feb 7;17(2):e0263072. doi: 10.1371/journal.pone.0263072 (PMC8820635; doi:10.1371/journal.pone.0263072)
Supplement: S1 File — (DOCX) [file pone.0263072.s001.docx]

**Section 1. Intro**

I’d like to start by asking a bit about you.

1. Tell me a little bit about yourself and your family?

***[Probe for age, job, family, etc.]***

**Section 2. Defining high quality of care**

I’m going to move now to asking questions about health care. Please think about when you or your family have been sick and need to seek medical care.

1. Where do you and your family usually seek health care services?
   1. Why do you choose to get care there?

Sometimes we receive health care services that are very good, sometimes very poor, and sometimes in between.

1. Please describe a recent time when you have received very good health care services from a doctor, nurse, or other health care provider.
   1. What made it a very good service?
2. Now please describe a recent time when you or your family received unacceptable or poor care from a health care provider.
   1. What made it poor care?
3. In your opinion, what do you think makes some healthcare services good and some bad?
4. Where do women in your community go to get antenatal care?
   1. What about contraceptive methods?
   2. How do you women in your community know where to get good care for these services?

**Section 3. Prior to abortion/MR care**

Now I would like to ask you some questions about your recent pregnancy.

1. What were your feelings or beliefs about [abortion/MR] in general before you decided to [have an abortion/seek MR] services?
2. What had you heard about [abortion/MR] before you got to care?
   1. What about safety or potential risks?
   2. What about consequences after the [abortion/MR]?

- 1. What about how you would be treated?
  2. What about the laws for when a woman can receive abortion/MR?

1. Where did you learn this information?
2. How did you decide to [have an abortion/seek MR] services?
3. Did you do anything to try and end the pregnancy in other ways before you contacted ***[insert name]***? Tell me about that.
4. How did you find out about ***[insert name]***?
   1. What had you heard about them?
5. What made you decide to seek services from ***[insert name]***?
   1. Did you hear stories about other women obtaining [abortion/MR] services that influenced your decision about where to go? Tell me about that.
6. What expectations did you have for how you would be treated when getting the [abortion/MR] care?
   1. What about fears? What was your biggest fear?
7. Did you speak to a health care provider about your pregnancy before you contacted ***[insert name]*** for the first time?

***[IF YES]***

1. Tell me about that
2. What did they tell you?
3. How did they treat you?
4. Can you estimate how far along were you in the pregnancy when you first contacted ***[insert name]?*** You can use your best guess if you are not sure.

**Section 4. During abortion/MR care**

I am curious to hear more about your experience with the counselor/health worker.

1. How were you treated during your interactions with ***[insert name]***?
   1. What made you feel that way?
2. Did anything they say scare you? Why?
3. In what ways did they make you feel prepared or unprepared for the abortion/MR?
4. What information did you receive from the counselor/health worker that was hard to understand, if any?
5. What did the helpline staff/health worker do to protect or not protect your privacy?
   1. How important was this for you?
6. At any point did you feel judged during your experience at ***[insert name]***? Tell me about that.
7. Did you have other any negative interactions with any staff/health workers? Tell me about that.
8. Tell me about the information you received about contraception after your abortion/MR service.
   1. Was there information that you wanted but did not receive related to contraception? Tell me about that.
9. Who was with you when you took the pills or had the procedure, if anyone? How was this person supportive or not supportive?
10. How many times did you talk to the counselor(s)/call center staff during the whole process?

**Section 5. After abortion/MR care**

1. Did you see a doctor or go to a clinic during or after [the abortion/ending the pregnancy]? Why or why not?

***[IF YES]***

1. What did you tell the provider about what happened?
2. What kind of medical treatment did you receive?
3. How did they treat you?
4. What was the best part of the care you received? Why?
5. What was the worst part of the care you received? Why?
6. Thinking back on the cost of the [abortion/MR], how would you describe it? How manageable was this for you?
7. Do you think your age affected how you were treated?
   1. Do you think the experience would have been the same or different for a friend of yours who is much older than you? Much younger than you? Why?
8. Do you think your marital status affected how you were treated?
   1. Do you think the experience would have been the same or different for a friend of yours who is **(*Married or unmarried)***  ***[Select the opposite experience of the participant]***

**Section 6. Reflecting on the abortion/MR experience**

1. If someone had never heard of ***[insert name]***, how would you describe it to them?
2. What would have made the experience with ***[insert name]*** better?
3. How did your actual experience getting [abortion/MR] care match or not match your expectations?
4. Have you had an [abortion/MR] experience before this recent pregnancy?
   1. How does this experience compare to your previous one?

***[Probe for: safety, pain, timeliness, and experience with provider]***

1. What advice would you give a friend who is also seeking to end her pregnancy? What should she know about good care?
2. If you had to describe the most important three parts of the best abortion/MR care, what three parts would you say? Please mention the parts that feel most important to you, no matter how big or small.

Lastly…..

1. What was it like to participate in this interview?
2. Before we end, I would like to ask you a few more questions about yourself. We ask these questions of everyone we speak with.
3. How old are you? _________
4. What is your relationship status right now?
5. How many children do you have, if any? ____________
6. Are you currently working? In school? Both?

**-- Thank you for taking the time to do this interview with us.--**
